# Comparing survival and treatment response of patients with acquired T790M mutation second‐line osimertinib versus sequential treatment of chemotherapy followed by osimertinib: A real‐world study

**DOI:** 10.1111/1759-7714.14198

**Published:** 2021-10-26

**Authors:** Chin‐Chou Wang, Chien‐Hao Lai, Yu‐Ping Chang, Huang‐Chih Chang, Chia‐Cheng Tseng, Kuo‐Tung Huang, Meng‐Chih Lin

**Affiliations:** ^1^ Divisions of Pulmonary and Critical Care Medicine, Department of Internal Medicine, Kaohsiung Chang Gung Memorial Hospital Chang Gung University College of Medicine Kaohsiung Taiwan; ^2^ Department of Respiratory Therapy, Kaohsiung Chang Gung Memorial Hospital Chang Gung University College of Medicine Kaohsiung Taiwan; ^3^ Department of Respiratory Care Chang Gung University of Science and Technology Chiayi Taiwan

**Keywords:** afatinib, EGFR T790M mutation, EGFR‐TKIs sequences, erlotinib, gefitinib, osimertinib

## Abstract

**Purpose:**

To investigate the survival benefit with first/second generation epidermal growth factor receptor tyrosine kinase inhibitors (EGFR‐TKIs) and osimertinib in different treatment sequences.

**Methods:**

We retrospectively screened 3807 patients diagnosed with cancer between 2013 and 2019 at Kaohsiung Chang Gung Memorial Hospital. In total, 76 patients with EGFR T790M mutation who received osimertinib after re‐biopsy or liquid biopsy were enrolled for the analysis.

**Results:**

The median progression‐free survival (PFS), median overall survival (OS), and median OS2 of the 76 patients were 11.93, 66.53, and 29.57 months, respectively. A significant difference was observed in the disease control rate between those who received osimertinib treatment after chemotherapy (group A) and those who received osimertinib immediately following EGFR‐TKI therapy (group B) (34 [94.4%] vs. 31 [77.5%], *p* = 0.036). In addition, chronic obstructive pulmonary disease tended to be a poor prognostic factor for PFS and OS.

**Conclusion:**

This real‐world analysis revealed that previous chemotherapy could affect the treatment outcomes of patients with non‐small cell lung cancer treated with osimertinib. Osimertinib treatment following first/second generation EGFR‐TKI treatment or chemotherapy resulted in improved survival benefit.

## Graphical abstract

Osimertinib treatment after one line of chemotherapy (group A) had a better response rate and a better PFS than osimertinib treatment immediately following treatment with first/second generation EGFR‐TKIs (group B). COPD tended to a poor prognostic factor for PFS and OS2 in patients with osimertinib use.

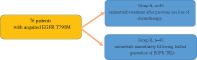

## INTRODUCTION

Lung cancer has a high prevalence and high mortality worldwide. Non‐small cell lung cancer (NSCLC) accounts for ~80%–85% of all lung cancer cases. Lung cancer treatment can be personalized using histological and molecular biology tests. Among various driver oncogenes, epidermal growth factor receptor (EGFR) mutations are the earliest and key genetic drivers of NSCLC. EGFR mutations are present in 10% of the Caucasian population and 40%–50% of the Asian population, which includes the Taiwanese population.^1^, ^2^, ^3^ Clinical trials and studies have shown that EGFR‐tyrosine kinase inhibitors (TKIs) produce better response rates and fewer adverse reactions than do platinum‐based chemotherapy regimens. The objective response rates of first/second generation EGFR‐TKIs have been 60%–80%, and the median progression‐free survival (PFS) durations have been 10–13 months.^4^, ^5^, ^6^, ^7^, ^8^, ^9^, ^10^, ^11^, ^12^ During disease progression (PD), newly acquired resistant EGFR p.Thr790Met (T790M) point mutations developed in 50%–70% of the patients.^13^, ^14^, ^15^ These acquired resistant mutations enhance the binding affinity of adenosine triphosphate to the EGFR kinase domain, thereby reducing the efficacy of the first/second generation EGFR‐TKIs.

Osimertinib, a third generation EGFR‐TKI, was designed, which is active in NSCLCs harboring the EGFR T790M mutation.^16^, ^17^, ^18^, ^19^ Furthermore, AURA3, a phase 3 clinical trial for investigating osimertinib, reported that osimertinib was associated with better PFS than standard chemotherapy for patients with NSCLC having acquired T790M mutations.^19^ Therefore, re‐biopsy or liquid biopsy is needed to confirm the mechanism of acquired drug resistance when patients with EGFR mutations develop PD after EGFR‐TKI treatment.

On the basis of European Society for Medical Oncology and National Comprehensive Cancer Network guidelines, osimertinib is strongly recommended for patients with acquired T790M after first/second generation EGFR‐TKIs. In Taiwan, gefitinib (since November 2007) was covered by national reimbursement before erlotinib (since June 2008) and afatinib (since May 2014). Furthermore, osimertinib was approved for second‐ and first‐line use in 2016 and 2019, respectively, but is covered by national reimbursement since April 2020 in Taiwan. Therefore, patients with acquired T790M, after first/second generation TKIs, underwent chemotherapy first instead of using third generation EGFR‐TKI during disease progression.

We conducted this retrospective study to evaluate the survival benefit of patients with acquired T790M mutation receiving second‐line osimertinib versus sequential treatment of chemotherapy followed by osimertinib.

## METHODS

**FIGURE 1:**
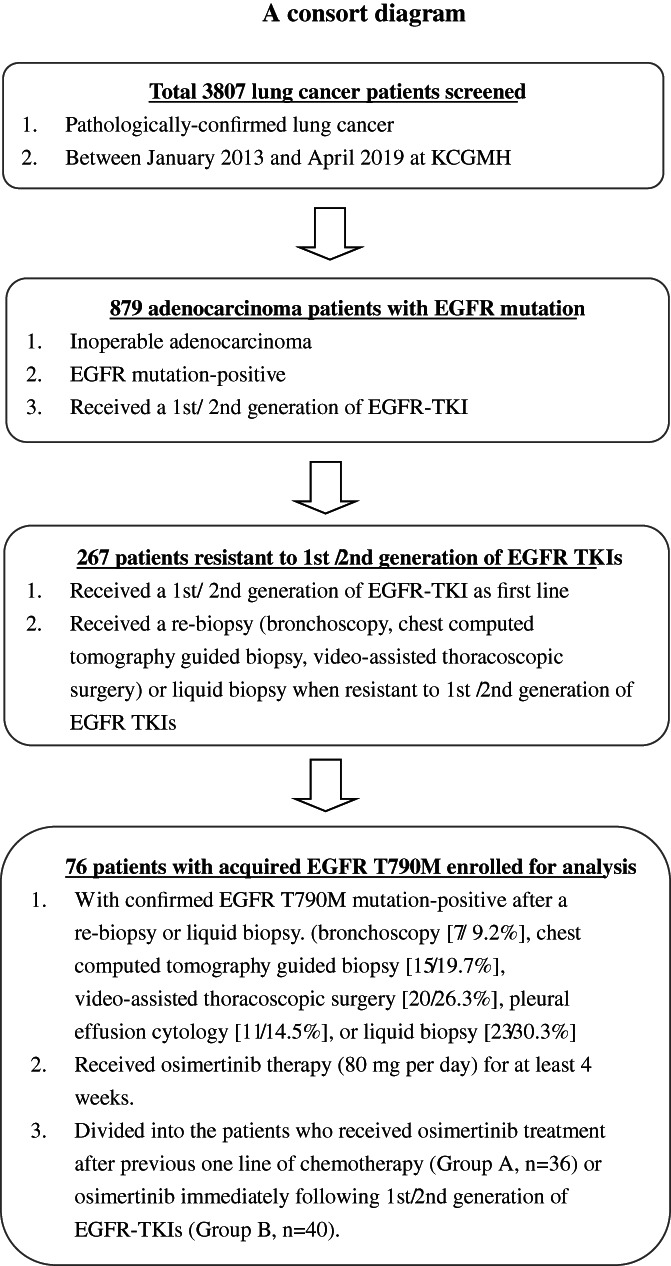
Flow chart describing the enrolment of patients in the study. EGFR, epidermal growth factor receptor; KCGMH, Kaohsiung Chang Gung Memorial Hospital; TKIs, tyrosine kinase inhibitors

The study retrospectively screened 3807 patients with pathologically confirmed lung cancer between January 2013 and April 2019 at Kaohsiung Chang Gung Memorial Hospital. Among these patients, 879 patients with inoperable EGFR mutation‐positive adenocarcinoma had received first generation EGFR‐TKI (gefitinib or erlotinib) or second generation EGFR‐TKI (afatinib) as the first‐line therapy. Furthermore, 267 of these 879 patients, who were resistant to first/second generation EGFR‐TKIs, had received re‐biopsy (including bronchoscopy, chest computed tomography‐guided biopsy, or video‐assisted thoracoscopic surgery) or liquid biopsy (the Department of Pathology of Kaohsiung Chang Gung Memorial Hospital was in charge of the detection of the EGFR T790M mutation in cell‐free plasma DNA) between March 2015 and December 2018. EGFR‐TKI resistance was defined as radiological progression based on Response Evaluation Criteria in Solid Tumors (RECIST) v1.121 or death. Among these patients, 76 patients with EGFR T790M mutation‐positive adenocarcinomas who had received osimertinib therapy (80 mg/day) for at least 4 weeks were enrolled for the analysis (Figure 1). These patients were divided into group A (patients who received osimertinib treatment after one line of chemotherapy; *n* = 36) and group B (patients who received osimertinib treatment immediately following treatment with another EGFR‐TKI; *n* = 40).

Each of these 76 patients received a chest computed tomography scan at osimertinib treatment initiation and at every 3 months thereafter to evaluate their tumor responses. Furthermore, brain magnetic resonance imaging and technetium 99m‐methyl diphosphonate bone scans were performed if related symptoms were observed. PFS, overall survival (OS), OS2 (defined as OS after the first line of first/second generation EGFR‐TKIs), overall response rate (ORR), and disease control rate (DCR) were calculated to evaluate their efficacy. PFS was calculated from osimertinib initiation until radiological progression based on RECIST v1.121 or death, with censoring at the last follow‐up if the patient did not experience PD. Complete response was defined as no detectable evidence of a tumor; partial response was defined as a decrease in tumor size; PD was defined as an increase in tumor size; and stable disease was defined as neither partial response nor PD based on RECIST v1.121 during osimertinib treatment. ORR was defined as the percentage of patients with complete response or partial response during osimertinib treatment, whereas the DCR was calculated as the percentage of patients who exhibited a complete response, partial response, or stable disease. Furthermore, the OS duration was calculated as the duration from osimertinib treatment initiation until patient death. Furthermore, outcomes were determined through a computed tomography scan before osimertinib initiation and immediately after treatment in this study.

### Statistical analysis

Data (including age, sex, tumor size, nodal stage, and EGFR mutation subtypes) were collected and analyzed using SPSS for Windows version 15.0 (SPSS). Quantitative variables are presented as average ± standard deviation. Statistical significance of the univariate analysis was determined using the Mann–Whitney *U* test for continuous variables and χ^2^ test for discontinuous variables.

The Kaplan–Meier method was used to estimate PFS and OS. Moreover, a Cox proportional hazards regression was performed to evaluate the determinants of PFS and OS. Differences were considered significant when the *p*‐value was <0.05.

## RESULTS

The demographic and clinical characteristics of 76 patients with EGFR T790M mutation–positive adenocarcinomas who received osimertinib therapy are described in Table 1. All patients had adenocarcinoma histology and were at an advanced stage of adenocarcinoma. In group A (*n* = 36), one line of chemotherapy was administered before osimertinib. Among these 36 patients, 35 (97.2%) patients were given pemetrexed with cisplatin/carboplatin and one (2.8%) patient was given docetaxel with carboplatin. Anti‐angiogenesis was not administered to any of these 36 patients. The mean age of the patients was 61.91 ± 10.49 (range = 36–81) years (group A: 62.61 ± 9.82 and group B: 61.28 ± 11.15); 30 (39.5%) patients were men and 46 (60.5%) were women (group A: 12/24 [33.3%/66.7%] and group B: 18/22 [45.0%/55.0%]).

**TABLE 1:** Demographic and clinical characteristics of all patients (*n* = 76)

		Total (*n* = 76)	Group A (*n* = 36, 47.4%)	Group B (*n* = 40, 52.6%)	*p*
Sex	Male	30 (39.5%)	12 (33.3%)	18 (45.0%)	0.211
	Female	46 (60.5%)	24 (66.7%)	22 (55.0%)	
Age (in years)		61.91 ± 10.49 (36–81)	62.61 ± 9.82 (44–80)	61.28 ± 11.15 (36–81)	0.583
	>65	29 (38.2%)	14 (38.9%)	15 (37.7%)	0.544
	≤65	47 (61.8%)	22 (61.1%)	25 (62.5%)	
BMI		24.66 ± 3.60 (16.31–33.06)	24.36 ± 3.33 (18.33–32.25)	24.95 ± 3.85 (16.31–33.06)	0.476
Tumor size (in cm)		4.55 ± 2.29 (0.8–15.0)	4.05 ± 1.50 (0.8–7.2)	5.00 ± 2.77 (1.0–15.0)	0.071
Stage	IIIB	1 (1.3%)	0	1 (1.3%)	0.526
	IV	75 (98.7%)	36 (100%)	39 (98.7%)	
First EGFR mutation test	Del 19	42 (55.3%)	21 (58.3%)	21 (52.5%)	0.390
	L858R	34 (44.7%)	15 (41.7%)	19 (47.5%)	
Second EGFR mutation test	Del 19 and T790M	42 (55.3%)	21 (58.3%)	21 (52.5%)	0.390
	L858R and T790M	34 (44.7%)	15 (41.7%)	19 (47.5%)	
ECOG	0	23 (30.3%)	11 (30.6%)	12 (30.0%)	0.312
	1	51 (67.1%)	23 (63.9%)	28 (70.0%)	
	2	2(2.6%)	2 (5.6%)	0 (%)	
	3–4	23 (30.3%)	11 (30.6%)	12 (30.0%)	
Comorbidities^a^	DM	8 (10.5%)	4 (11.1%)	4 (10.0%)	0.583
	COPD	9 (11.8%)	4 (11.1%)	5 (12.5%)	0.568
	Hypertension	18 (23.7%)	8 (22.2%)	10 (25.0%)	0.495
Smoking	Never	59 (77.6%)	27 (75.0%)	32 (80.0%)	0.615
	Ever	11 (14.5%)	5 (13.9%)	6 (15.0%)	
	Current	6 (7.9%)	4 (11.1%)	2 (5.0%)	
CEA level	≤5	25 (32.9%)	13 (36.1%)	12 (30.0%)	0.374
	>5	51 (67.1%)	23 (63.9%)	28 (70.0%)	
Time interval between biopsies^b^ (in months)		24.19 ± 16.88 (1.33–99.10)	26.21 ± 15.75 (3.53–77.60)	22.38 ± 17.84 (1.33–99.10)	0.327
Time interval between first PD and re‐biopsy/liquid biopsy^c^ (in days)		8.67 ± 2.30 (7–17)	8.56 ± 2.78 (4–17)	8.77 ± 1.79 (7–14)	0.688
First‐line EGFR TKIs	Geftinib	36 (47.4%)	21 (58.3%)	15 (37.5%)	0.078
	Erlotinib	21 (27.6%)	10 (27.8%)	11 (27.5%)	
	Afatinib	19 (25.0%)	5 (13.9%)	14 (35.0%)	
Osimertinib response	CR	0 (0%)			
	PR	40 (52.6%)			
	SD	25 (32.9%)			
	PD	11 (14.5%)			
Osimertinib therapy PFS total (median, in months)		11.93			
OS (median, in months)		66.53			
OS2 (median, in months)		29.57			

*Note*: Group A consisted of patients who received osimertinib treatment after previous chemotherapy. Group B consisted of patients who received osimertinib immediately after treatment with first/second generation EGFR‐TKIs.

Abbreviations: CEA, carcinoembryonic antigen, defined when first/second EGFR‐TKIs disease progression at first line; COPD, chronic obstructive pulmonary disease; CR, complete response; DM, diabetes mellitus; ECOG, Eastern Cooperative Oncology Group Performance score, defined when first/second EGFR‐TKIs disease progression at first line; OS, median overall survival; OS2, median overall survival after the first line of first/second generation EGFR‐TKI treatment; PD, progression disease; PFS, median progression‐free survival; PR, partial response; SD, stable disease.

^a^ Other comorbidities included stroke (0%), renal impairment (3, 3.9%), and hepatic impairment (2, 2.6%).

^b^ Time interval between biopsies defined as between first biopsy when lung cancer diagnosed and re‐biopsy or liquid biopsy when disease progression with first/second generation EGFR‐TKIs.

^c^ Time interval between first PD and re‐biopsy/liquid biopsy defined as the time when disease progression after first line with first/second generation EGFR‐TKIs to re‐biopsy/liquid biopsy.

EGFR genotyping at the initial diagnosis showed Del 19 and L858R mutations in 42 (55.3%) and 34 (44.7%) of these patients with adenocarcinoma. All patients were pretreated with EGFR‐TKIs: 36 (47.4%), 21 (27.6%), and 19 (25.0%) received gefitinib, erlotinib, and afatinib, respectively. EGFR genotyping at the secondary (re‐biopsy or liquid biopsy) diagnosis showed Del 19 combined with T790M in 42 (55.3%) patients and L858R combined with T790M in 34 (44.7%) patients. The time interval between biopsies was 24.19 ± 16.88 (1.33–99.10) months (group A: 26.21 ± 15.75 and group B: 22.38 ± 17.84).

Of the 76 patients, 40 (52.6%), 25 (32.9%), and 11 (14.5%) exhibited partial responses, stable diseases, and PD, respectively. The ORR was 52.6%, and the DCR was 85.5%. Furthermore, their median PFS, median OS, and median OS2 were 11.93, 66.53, and 29.57 months, respectively.

Table 2 demonstrates responses to osimertinib treatment after first‐line therapy with different EGFR‐TKIs. No significant difference was observed in response to osimertinib between patients with first‐line treatment with different EGFR‐TKIs. The median PFS values of those who received osimertinib therapy after first‐line therapy with gefitinib, afatinib, or erlotinib were 12.83, 11.93, and 10.9 months, respectively (*p* = 0.424). The median OS values of those who received osimertinib therapy after first‐line therapy with gefitinib, afatinib, or erlotinib were 87.93, 49.00, and 42 months, respectively; furthermore, no significant difference was observed in OS between patients treated with different EGFR‐TKIs as first‐line treatment (*p* = 0.484), but OS seemed to be longer in the gefitinib group (87.93 months). The median OS2 values of those who received osimertinib therapy after first‐line therapy with gefitinib, afatinib, or erlotinib were 22.73, 16.1, and 34 months, respectively; furthermore, no significant difference in OS was observed between patients treated with the different EGFR‐TKIs as first‐line treatment (*p* = 0.095).

**TABLE 2:** Response to osimertinib treatment after previous therapy with a different EGFR‐TKI as first‐line treatment (*n* = 76)

Group	Geftinib (*n* = 36, 47.4%)	Afatinib (*n* = 19, 25.0%)	Erlotinib (*n* = 21, 27.6%)	*p*‐value
PR	21 (58.3%)	8 (42.1%)	11 (52.4%)	0.839
SD	10 (27.8%)	8 (42.1%)	7 (33.3%)	
PD	5 (13.9%)	3 (15.8%)	3 (14.3%)	
PFS (in months)	12.83	11.93	10.9	0.424
OS (in months)	87.93	49.00	42.0	0.203
OS2 (in months)	23.73	16.1	34.0	0.095

*Note*: Pearson's χ^2^ test.

Abbreviations: OS, median overall survival; OS2, median overall survival after first‐line treatment with first/second generation EGFR‐TKIs; PD, progression disease; PFS, median progression‐free survival; PR, partial response; SD, stable disease.

Table 3 presents the response, PFS, OS, and OS2 of groups A and B. No significant difference was observed in the ORR between these two groups (63.9% vs. 42.5%, *p* = 0.063), but a significant difference was observed in the DCR between these two groups (94.4% vs. 77.5%, *p* = 0.036). No significant difference was observed in PFS (15.7 vs. 10.83 months, *p* = 0.248), OS (49.00 vs. NA months, *p* = 0.430), and OS2 (25.43 vs. NA months, *p* = 0.933) between these two groups.

**TABLE 3:** Response, PFS, OS, and OS2 of patients who received osimertinib treatment after previous chemotherapy (group A) or immediately following treatment with first/second generation EGFR‐TKIs (group B) (*n* = 76)

Group	Group A (*n* = 36, 47.4%)	Group B (*n* = 40, 52.6%)	*p*‐value
PR	23 (63.9%)	17 (42.5%)	0.063
SD	11 (30.6%)	14 (35.0%)	
PD	2 (5.6%)	9 (22.5%)	
DCR	34 (94.4%)	31 (77.5%)	0.036
PD	2 (5.6%)	9 (22.5%)	
PFS (in months)	15.7	10.83	0.248
	HR = 0.717 (0.41–1.26)		
OS (in months)	49.00	NA	0.430
	HR = 1.348 (0.64–2.84)		
OS2 (in months)	25.43	NA	0.933
	HR = 1.033 (0.484–2.202)		

*Note*: Pearson's χ^2^ test. Group A consisted of patients who received osimertinib treatment after previous chemotherapy. Group B consisted of patients who received osimertinib immediately following treatment with first/second generation EGFR‐TKIs.

Abbreviations: DCR, disease control rate; OS, median overall survival; OS2, median overall survival after first‐line treatment with first/second generation EGFR‐TKIs; PD, progression disease; PFS, median progression‐free survival; PR, partial response; SD, stable disease.

Table 4 presents the subgroup analysis of PFS, OS, and OS2. In terms of PFS, significant differences were observed in patients ≥1 months after EGFR‐TKI treatment (*p* = 0.042, hazard ratio [HR] = 0.55, 95% confidence interval [CI] = 0.31–0.98), with a low BMI (body mass index) level (*p* = 0.042, HR = 0.73, 95% CI = 0.54–0.99), and without chronic obstructive pulmonary disease (COPD) (*p* = 0.005, HR = 0.36, 95% CI = 0.17–0.75). In terms of OS, a significant difference was observed only in patients without COPD (*p* = 0.043, HR = 0.42, 95% CI = 0.18–0.99). In terms of OS2, a significant difference was observed only in the patients without COPD (*p* = 0.008, HR = 0.33, 95% CI = 0.14–0.78). We select parameters which *p* < 0.1 to enter multiple analysis. Using a Cox proportional hazards regression, we determined, COPD tended to be a poor prognostic factor for PFS and OS2 (Table 5).

**TABLE 4:** Median PFS, OS, and OS2 of patients with T790M‐mutated lung adenocarcinoma (subgroup analysis; *n* = 76)

Group			*p*‐value	Hazard ratio (95% CI)
Months after receiving a previous EGFR‐TKI	<1 month (*n* = 35, 46.0%)	≥1 month (*n* = 41, 54.0%)		
PFS (in months)	10.30	15.70	0.042	0.55 (0.31–0.98)
OS (in months)	NA	61.0	0.696	0.86 (0.40–1.83)
OS2 (in months)	NA	29.57	0.220	0.62 (0.28–1.34)
BMI level	<27 (*n* = 57, 75.0%)	≥27 (*n* = 19, 25.0%)		
PFS (in months)	12.57	9.50	0.042	0.73 (0.54–0.99)
OS (in months)	NA	42.00	0.061	0.70 (0.48–1.03)
OS2 (in months)	32.00	22.93	0.098	0.73 (0.50–1.07)
CEA level	≤5 (*n* = 25, 32.9%)	>5 (*n* = 51, 67.1%)		
PFS (in months)	11.93	12.13	0.652	0.87 (0.48–1.59)
OS (in months)	65.53	61.00	0.934	1.03 (0.47–2.23)
OS2 (in months)	34.10	29.56	0.806	0.91 (0.42–1.95)
DM	Without (*n* = 68, 89.5%)	With (*n* = 9, 10.5%)		
PFS (in months)	12.43	7.90	0.284	0.63 (0.27–1.48)
OS (in months)	61.00	46.43	0.404	0.66 (0.25–1.75)
OS2 (in months)	32.00	23.57	0.222	0.55 (0.21–1.46)
HTN	Without (*n* = 58, 76.3%)	With (*n* = 18, 23.7%)		
PFS (in months)	12.43	9.67	0.620	0.85 (0.44–1.63)
OS (in months)	66.53	61.00	0.901	0.95 (0.42–2.17)
OS2 (in months)	29.57	51.53	0.595	0.80 (0.36–1.81)
COPD	Without (*n* = 67, 88.2%)	With (*n* = 9, 11.8%)		
PFS (in months)	12.43	4.30	0.005	0.36 (0.17–0.75)
OS (in months)	66.53	30.60	0.043	0.42 (0.18–0.99)
OS2 (in months)	32.00	19.43	0.008	0.33 (0.14–0.78)
Initial brain metastasis	Without (*n* = 68, 89.5%)	With (*n* = 8, 10.5%)		
PFS (in months)	11.93	12.57	0.773	0.86 (0.31–2.41)
OS (in months)	66.53	NA	0.791	0.85 (0.26–2.83)
OS2 (in months)	29.56	23.63	0.329	0.55 (0.16–1.87)
Brain metastasis (osi)	Without (*n* = 55, 72.4%)	With (*n* = 21, 27.6%)		
PFS (in months)	12.83	10.90	0.196	0.81 (0.59–1.12)
OS (in months)	66.53	41.27	0.170	0.76 (0.51–1.13)
OS2 (in months)	32.00	23.63	0.102	0.72 (0.48–1.08)

*Note*: Brain metastasis (OSI) = brain metastasis was noted before osimertinib treatment.

Abbreviations: BMI, body mass index (obesity is defined as BMI > 27 in Taiwan); CEA, carcinoembryonic antigen, defined when first/second generation EGFR‐TKI disease progression at first line; COPD, chronic obstructive pulmonary disease; DM, diabetes mellitus; HTN, hypertension; OS, median overall survival; OS2, median overall survival after first‐line treatment with first/second generation EGFR‐TKIs; PFS, median progression‐free survival.

**TABLE 5:** Cox regression analysis: effects of potential prognostic factors on PFS, OS, and OS2 for T790M‐mutated lung adenocarcinoma

Prognostic factor		Univariate analysis		Multivariate analysis	
		Hazard ratio	*p*‐value	Hazard ratio	*p*‐value
PFS					
Months after receiving a previous EGFR‐TKI	≥1 m vs. <1 m	0.55 (0.31–0.98)	0.042	0.64 (0.35–1.18)	0.153
BMI level	<27 vs. ≥27	0.73 (0.54–0.99)	0.042	0.61 (0.32–1.13)	0.116
COPD	No vs. Yes.	0.36 (0.17–0.75)	0.005	0.43 (0.20–0.91)	0.030
OS					
BMI level	<27 vs. ≥27	0.70 (0.48–1.03)	0.061	0.57 (0.26–1.25)	0.163
COPD	No vs. Yes.	0.42 (0.18–0.99)	0.043	0.50 (0.20–1.23)	0.133
OS2					
BMI level	<27 vs. ≥27	0.73 (0.50–1.07)	0.098	0.57 (0.26–1.25)	0.124
COPD	No vs. Yes.	0.33 (1.41–0.78)	0.008	0.50 (0.20–1.23)	0.014

Abbreviations: COPD, chronic obstructive pulmonary disease; OS, median overall survival; PFS, median progression‐free survival.

All patients with brain metastasis were noted to have solid tumor before osimertinib treatment in this study. As shown in Table 6, in group A, 10 (27.8%) patients had brain metastasis before osimertinib; the PFS of patients with brain metastasis before osimertinib was 11.07 months and of those without brain metastasis before osimertinib was 16.90 months. In group B, 11 (27.5%) patients had brain metastasis before osimertinib; the PFS of patients with brain metastasis before osimertinib was 10.27 months and of those without brain metastasis before osimertinib was 11.37 months. Therefore, PFS was better with osimertinib as the third‐line treatment than as the second‐line treatment.

**TABLE 6:** PFS, OS, and OS2 of brain metastasis before osimertinib subgroup analysis in group A and group B (*n* = 76)

Brain metastasis (OSI)	No	Yes	*p*‐value	Hazard ratio (95% CI)
In group A				
Total *n* = 36	26 (72.2%)	10 (27.8%)		
PFS (months)	16.90	11.07	0.168	0.70 (0.42–1.18)
OS (months)	61.00	36.23	0.005	0.45 (0.24–0.83)
OS2 (months)	32.00	23.63	0.023	0.55 (0.32–0.95)
In group B				
Total *n* = 40	29 (72.5%)	11 (27.5%)		
PFS (months)	11.37	2.57	0.340	0.81 (0.53–1.25)
OS (months)	NA	NA	–	–
OS2 (months)	NA	NA	–	–

*Note*: Group A consisted of patients who received osimertinib treatment after previous chemotherapy. Group B consisted of patients who received osimertinib immediately following treatment with first/second generation EGFR‐TKIs. Brain metastasis (OSI) = brain metastasis was noted before osimertinib treatment. All patients with brain metastasis were noted to have solid tumor type before osimertinib treatment.

Abbreviations: OS, median overall survival; OS2, median overall survival after first‐line treatment with first/second generation EGFR‐TKIs; PFS, median progression‐free survival.

Table 7 presents the survival difference between L858R and Del19 in our study. Among L858R‐positive patients, no significant difference was observed in PFS between groups A and B (12.57 months vs. 9.00 months, *p* = 0.189). Furthermore, in Del19‐positive patients, no significant difference was observed in PFS between groups A and B (21.13 months vs. 11.87 months, *p* = 0.884). Nevertheless, group A showed long PFS in both L858R‐ and Del19‐positive patients.

**TABLE 7:** PFS, OS, and OS2 of patients who received osimertinib treatment after previous chemotherapy (group A) or immediately following treatment with first/second generation EGFR‐TKIs (group B)

Group *n* = 76	Group A	Group B	*p*‐value	Hazard ratio (95% CI)
L858R	15 (41.7%)	19 (47.5%)	0.390	
Del 19	21 (58.3%)	21 (52.5%)		
L858R *n* = 34	15 (14.1%)	19 (55.9%)		
PFS (months)	12.57	9.00	0.189	0.76 (0.51–1.15)
OS (months)	66.53	NA	0.932	0.98 (0.58–1.64)
OS2 (months)	25.4	NA	0.604	0.86 (0.51–1.48)
Del 19 *n* = 42	21 (50.0%)	21 (50.0%)		
PFS (months)	21.13	11.87	0.884	0.97 (0.64–1.46)
OS (months)	47.60	NA	0.238	1.41 (0.79–2.55)
OS2 (months)	25.43	NA	0.484	1.23 (0.69–2.21)
Total *n* = 76	36, 47.4%	40, 52.6%		

*Note*: L858R and Del 19 subgroup analysis (*n* = 76). Group A consisted of patients who received osimertinib treatment after previous chemotherapy. Group B consisted of patients who received osimertinib immediately following treatment with first/second generation EGFR‐TKIs.

Abbreviations: OS, median overall survival; OS2, median overall survival after first‐line treatment with first/second generation EGFR‐TKIs; PFS, median progression‐free survival.

## DISCUSSION

In this study, we evaluated the response of patients with NSCLC with T790M EGFR–resistant mutations to osimertinib following treatment with first/second generation EGFR‐TKIs. We found that (1) the gefitinib group had better OS compared with the other EGFR‐TKI treatment groups (Table 2), (2) osimertinib treatment following chemotherapy (group A) had a better response rate and PFS than group B (Table 3), and (3) brain metastasis during osimertinib treatment was a poor prognostic factor for PFS.

In LUX‐Lung 3 and LUX‐Lung 6 trials, OS was significantly longer for patients with EGFR Del19‐positive tumors in the afatinib group than in the chemotherapy group: in LUX‐Lung 3, median OS was 33.3 months (95% CI = 26.8–41.5) in the afatinib group versus 21.1 months (16.3–30.7) in the chemotherapy group (HR = 0.54, 95% CI = 0.36–0.79, *p* = 0.0015); in LUX‐Lung 6, it was 31.4 months (95% CI = 24.2–35.3) versus 18.4 months (14.6–25.6), respectively (HR = 0.64, 95% CI = 0.44–0.94, *p* = 0.023). By contrast, no significant differences were observed based on treatment among patients with EGFR L858R–positive tumors in either trial. In LUX‐Lung 3, median OS was 27.6 months (19.8–41.7) in the afatinib group versus 40.3 months (24.3–not estimable) in the chemotherapy group (HR = 1.30, 95% CI = 0.80–2.11, *p* = 0.29); in LUX‐Lung 6, it was 19.6 months (95% CI = 17.0–22.1) versus 24.3 months (19.0–27.0), respectively (HR = 1.22, 95% CI = 0.81–1.83, *p* = 0.34). The absence of an effect on patients with L858R mutations suggests that EGFR Del19–positive disease might be distinct from EGFR L858R–positive disease.^6^, ^20^, ^21^ Furthermore, the different effects of EGFR‐TKIs between Del19 and L858R could explain why osimertinib as the third‐line treatment had better PFS than osimertinib as the second‐line treatment in our study. Furthermore, the results of LUX‐Lung 3 suggested that cisplatin plus pemetrexed promoted longer PFS in L858R patients (8.1 months) than in Del19 patients (5.6 months). Furthermore, another Japanese study results suggested that cisplatin plus pemetrexed regimen might confer higher efficacy for L858R patients in the second line or later settings.^22^ Therefore, chemotherapy has better survival benefit in L858R‐positive than in Del19‐positive patients.

In the AURA3 study,^19^ patients with acquired resistance to first/second generation EGFR‐TKIs (gefitinib, erlotinib, or afatinib) were randomized to osimertinib and platinum‐pemetrexed groups. The PFS of the osimertinib group was 10.1 months. In our study, the median PFS values of those who received osimertinib therapy after a first‐line therapy with gefitinib, afatinib, or erlotinib were 12.57, 11.87, and 11.37 months, respectively; they were slightly longer than in the AURA3 study. This difference in PFS may be because of differences in the assessment duration between AURA3 and our study. Assessments were performed every 6 weeks in the AURA3 study, whereas they were performed every 3 months in our study.

After pretreatment with gefitinib, osimertinib tended to have a better PFS in this study (Table 2). These data were similar to those of another study from Taiwan,^23^ in which PFS values for patients treated with first and second generation EGFR‐TKIs were 20.3 and 11.6 months, respectively (HR = 0.40, 95% CI = 0.18–0.82, *p* = 0.031).^23^ Kuo et al.^23^ used digital PCR in the re‐biopsy of tissues to determine the differences between the allele frequencies of mEGFR (19del or L858R; AF_mEGFR_) and T790M (AF_T790M_) after acquiring resistance between the first and second generation EGFR treatment. In the study by Kuo et al,^23^ the AF_T790M_/AF_mEGFR_ ratio of the first generation EGFR‐TKI treatment group was significantly higher than that of the second generation EGFR‐TKI treatment group. In addition, a highly significant correlation was observed between AF_T790M_ and AF_mEGFR_. This could explain why osimertinib tended to have a better PFS following pretreatment with gefitinib than with afatinib in this study. In our study, these data regarding the AF_T790M_/AF_mEGFR_ ratio were unavailable because of its retrospective nature. Therefore, the data from Kuo et al.^23^ cannot explain a better PFS following pretreatment with gefitinib than with erlotinib.

In Taiwan, gefitinib (since November 2007) was covered by national reimbursement earlier than erlotinib (since June 2008) and afatinib (since May 2014). Furthermore, osimertinib was approved for second‐ and first‐line use in 2016 and 2019, respectively, but has been covered by national reimbursement since April 2020 in Taiwan. The difference in the timing of approval and national reimbursement may have affected outcomes between these three first‐line EGFR‐TKIs. At least one line of chemotherapy before osimertinib was administered to 21 (58.3%) patients in the gefitinib group (group A), >10 (27.8%) patients in erlotinib group, and 5 (13.9%) patients in afatinib group. Furthermore, because of difference in the timing of approval and national reimbursement, the enrolled patients seemed to have an extra survival period due to chemotherapy before they received osimertinib.

Compared with first generation EGFR‐TKIs, second generation EGFR‐TKIs exhibited a broader inhibition spectrum and have an irreversible effect on the tyrosine kinases of EGFR and other ErbB family members.^24^ Investigations^25^, ^26^, ^27^ have shown that tumors resistant to second generation EGFR‐TKIs usually show undetectable levels of EGFR and HER2 amplification, which may indicate a greater advantage of activating EGFR mutant clones in tumors. By contrast, in tumors that acquired resistance to the first generation EGFR‐TKI, EGFR and HER2 amplification were found at a consistent frequency,^13^, ^28^ suggesting a less dominant place of EGFR‐activating mutations in this scenario.

Studies by Oxnard and Remon that examined the ratio of T790M to activating EGFR‐mutation alleles yielded controversial results regarding the predictive role of the T790M allele in liquid biopsies.^29^, ^30^ Oxnard et al.^29^ showed that the ratio of T790M to activating EGFR mutations is related to response depth to osimertinib treatment, whereas this association was not noted by Remon et al.^30^ in a similar study setting. Instead of liquid biopsy samples, our study showed that using tissue re‐biopsies (liquid biopsy:tissue re‐biopsy = 30.3%:69.7%) (Figure 1) is feasible for determining the predictive role of the T790M allele.

In our study, a trend toward a significant difference in the median PFS was observed between osimertinib use as second‐ and third‐line therapy. These PFS data were different from those of another study from Taiwan^23^ in which the HR was 1.03 (95% CI = 0.44–2.20, *p* = 0.941). Furthermore, PFS data in our study were different from those of the AURA2 study^31^; in that study, the PFS for osimertinib as second‐ and third‐line therapies were 11.0 (6.7–NR) and 12.4 (9.5–15.5) months, respectively. Furthermore, no significant difference was observed in OS between these studies. Moreover, many related studies have described the effects of second‐line EGFR‐TKI after the initial exposure^32^, ^33^, ^34^, ^35^, ^36^; furthermore, these studies have described different results. During chemotherapy, the original EGFR‐dependent cells may re‐grow, and a second remission may be obtained through the introduction of EGFR‐TKIs after chemotherapy. In addition to sensitivity to acquired T790M mutations, osimertinib is sensitive to original EGFR mutations (Del 19 and L8585R).^16^ This hypothesis may explain the increased RR and DCR in this study (Table 3) compared with other study results for second‐round EGFR‐TKIs of different designs.^32^, ^33^, ^34^, ^35^

To detect T790M resistance mutations, in most studies, re‐biopsy was performed when the disease progressed,^13^, ^37^ and the results have shown that T790M accounted for 50%–60% of the resistance mechanism. Because the cancer genome is heterogeneous, it can evolve over time, and it can interact with different treatments.^38^ It is unclear whether the timing of a re‐biopsy or liquid biopsy will affect the detection rate of T790M. However, in one study,^39^ the results suggested that no significant association exists between the re‐biopsy timing and T790M detection rate. In addition, this study showed that T790M can exist for a long time after the progression of EGFR‐TKI treatment, and it is an important carcinogenic driving factor.

Gefitinib (since November 2007) was covered by national reimbursement earlier than erlotinib (since June 2008) and afatinib (since May 2014) in Taiwan. This could explain why the gefitinib group had a longer PFS than the erlotinib and afatinib groups. Furthermore, osimertinib was approved for second‐line use since 2016 and first‐line use since 2019, but covered by national reimbursement since April 2020. The difference in the timing of approval and national reimbursement could affect outcome between these three first‐line EGFR‐TKIs.

Different tumor cells exhibit different morphological and phenotypic characteristics, including cell morphology, gene expression, metabolism, motility, proliferation, and metastatic potential. This phenomenon called tumor heterogeneity occurs in both intertumoral and intratumoral cells. This heterogeneity might result in a non‐uniform distribution of genetically distinct tumor‐cell subpopulations across and within disease sites (spatial heterogeneity) or temporal variations in the molecular makeup of cancer cells (temporal heterogeneity). Tumor heterogeneity could explain resistance to cancer therapies.^40^ Chemotherapy followed by osimertinib benefit could be explained by tumor heterogeneity. Furthermore, tumor heterogeneity could be used to explain why some patients with EGFR‐mutation did not get survival benefit when they received EGFR‐TKIs. For instance, EGFR coexisting with TP53 mutations contributed to poor prognosis in patients with adenocarcinoma.^41^

The patients in group A may be stronger than those in group B. During chemotherapy treatment in group A, the patients who were fragile may have been lost to follow‐up or died during the chemotherapy treatment.

NSCLC is the main cause of brain metastases.^42^, ^43^ Among recurrent/advanced NSCLC, brain metastases are a common cause of cancer‐related morbidity and mortality. As targeted therapy continues to improve the prognosis of NSCLC patients with target oncogene,^8^ the deterrence of brain metastases has become an increasingly relevant treatment problem. First‐ and second generation EGFR‐TKIs (i.e., gefitinib, erlotinib, and afatinib) cannot effectively cross the intact complete blood–brain barrier where the ratio of the patient's cerebrospinal fluid to plasma is as low as 0.01:0.003. In the AURA3 and FLAURA studies,^19^, ^44^ the PFS benefit of osimertinib was observed in patients with or without known or treated brain metastases at trial entry. Patients with brain metastases tended to have a worse PFS benefit (PFS = 15.2, 95% CI = 12.1–21.4 months) than those without brain metastases (PFS = 19.1, 95% CI = 15.2–23.5 months) in EGFR‐mutation NSCLC patients in the FLAURA study.^44^ This could explain why initial brain metastasis did not influence the osimertinib PFS, but brain metastasis during osimertinib treatment did influence the osimertinib PFS in our study.

This retrospective study has several limitations. First, this study was conducted at a single medical center, and therefore, the patient population may be biased by patient selection and referral patterns. Second, this study was a retrospective survey, which not only resulted in incomplete data for some patients, but also did not control for laboratory examinations. Third, the multiple lines of treatment before osimertinib administration may have confounded the effects. Fourth, some results showed a low *p*‐value, but not <0.05, which could be because of the small sample size. Another limitation was that any genomic alteration beyond EGFR mutations was not measured in this study. Only first generation EGFR‐TKIs were used for analysis in the AURA3 trial. Although both first and second generation EGFR‐TKIs were used for the analysis, it still was a retrospective analysis. In the future, further randomized controlled trial should be conducted to evaluate PFS and OS benefit between different sequences of EKFR‐TKIs.

## CONCLUSION

We observed that osimertinib treatment after one line of chemotherapy (group A) had a better response rate and a better PFS than osimertinib treatment immediately following treatment with first/second generation EGFR‐TKIs (group B). Osimertinib is neither easily available nor covered by national reimbursement in many countries. In our study, an alternative treatment sequence of chemotherapy followed by osimertinib had a better PFS benefit.

## CONFLICT OF INTEREST

The authors declare no conflicts of interest.

## References

[1] Bulbul A , Husain H . First‐line treatment in EGFR mutant non‐small cell lung cancer: is there a best option? Front Oncol. 2018;8:94. 29755953 10.3389/fonc.2018.00094 PMC5932412

[2] Kohno T , Nakaoku T , Tsuta K , Tsuchihara K , Matsumoto S , Yoh K , et al. Beyond ALK‐RET, ROS1 and other oncogene fusions in lung cancer. Transl Lung Cancer Res. 2015;4(2):156–64. 25870798 10.3978/j.issn.2218-6751.2014.11.11 PMC4384213

[3] Shi Y , Au JS , Thongprasert S , Srinivasan S , Tsai CM , Khoa MT , et al. A prospective, molecular epidemiology study of EGFR mutations in Asian patients with advanced non‐small‐cell lung cancer of adenocarcinoma histology (PIONEER). J Thorac Oncol. 2014;9(2):154–62. 24419411 10.1097/JTO.0000000000000033 PMC4132036

[4] Herbst RS , Morgensztern D , Boshoff C . The biology and management of non‐small cell lung cancer. Nature. 2018;553(7689):446–54. 29364287 10.1038/nature25183

[5] Thomas A , Liu SV , Subramaniam DS , Giaccone G . Refining the treatment of NSCLC according to histological and molecular subtypes. Nat Rev Clin Oncol. 2015;12:511. 25963091 10.1038/nrclinonc.2015.90

[6] Sequist LV , Yang JC , Yamamoto N , O'Byrne K , Hirsh V , Mok T , et al. Phase III study of afatinib or cisplatin plus pemetrexed in patients with metastatic lung adenocarcinoma with EGFR mutations. J Clin Oncol. 2013;31(27):3327–34. 23816960 10.1200/JCO.2012.44.2806

[7] Mitsudomi T , Morita S , Yatabe Y , Negoro S , Okamoto I , Tsurutani J , et al. Gefitinib versus cisplatin plus docetaxel in patients with non‐small‐cell lung cancer harbouring mutations of the epidermal growth factor receptor (WJTOG3405): an open label, randomised phase 3 trial. Lancet Oncol. 2010;11(2):121–8. 20022809 10.1016/S1470-2045(09)70364-X

[8] Rosell R , Carcereny E , Gervais R , Vergnenegre A , Massuti B , Felip E , et al. Erlotinib versus standard chemotherapy as first‐line treatment for European patients with advanced EGFR mutation‐positive non‐small‐cell lung cancer (EURTAC): a multicentre, open‐label, randomised phase 3 trial. Lancet Oncol. 2012;13(3):239–46. 22285168 10.1016/S1470-2045(11)70393-X

[9] Zhou C , Wu YL , Chen G , Feng J , Liu XQ , Wang C , et al. Erlotinib versus chemotherapy as first‐line treatment for patients with advanced EGFR mutation‐positive non‐small‐cell lung cancer (OPTIMAL, CTONG‐0802): a multicentre, open‐label, randomised, phase 3 study. Lancet Oncol. 2011;12(8):735–42. 21783417 10.1016/S1470-2045(11)70184-X

[10] Lynch TJ , Bell DW , Sordella R , Gurubhagavatula S , Okimoto RA , Brannigan BW , et al. Activating mutations in the epidermal growth factor receptor underlying responsiveness of non‐small‐cell lung cancer to gefitinib. N Engl J Med. 2004;350(21):2129–39. 15118073 10.1056/NEJMoa040938

[11] Maemondo M , Inoue A , Kobayashi K , Sugawara S , Oizumi S , Isobe H , et al. Gefitinib or chemotherapy for non‐small‐cell lung cancer with mutated EGFR. N Engl J Med. 2010;362(25):2380–8. 20573926 10.1056/NEJMoa0909530

[12] Paez JG , Jänne PA , Lee JC , Tracy S , Greulich H , Gabriel S , et al. EGFR mutations in lung cancer: correlation with clinical response to gefitinib therapy. Science. 2004;304(5676):1497–500. 15118125 10.1126/science.1099314

[13] Helena AY , Arcila ME , Rekhtman N , Sima CS , Zakowski MF , Pao W , et al. Analysis of tumor specimens at the time of acquired resistance to EGFR‐TKI therapy in 155 patients with EGFR‐mutant lung cancers. Clin Cancer Res. 2013;19(8):2240–7. 23470965 10.1158/1078-0432.CCR-12-2246 PMC3630270

[14] Ohashi K , Maruvka YE , Michor F , Pao W . Epidermal growth factor receptor tyrosine kinase inhibitor‐resistant disease. J Clin Oncol. 2013;31(8):1070–80. 23401451 10.1200/JCO.2012.43.3912 PMC3589701

[15] Kobayashi S , Boggon TJ , Dayaram T , Jänne PA , Kocher O , Meyerson M , et al. EGFR mutation and resistance of non‐small‐cell lung cancer to gefitinib. N Engl J Med. 2005;352(8):786–92. 15728811 10.1056/NEJMoa044238

[16] Cross DA , Ashton SE , Ghiorghiu S , Eberlein C , Nebhan CA , Spitzler PJ , et al. AZD9291, an irreversible EGFR TKI, overcomes T790M‐mediated resistance to EGFR inhibitors in lung cancer. Cancer Discov. 2014;4(9):1046–61. 24893891 10.1158/2159-8290.CD-14-0337 PMC4315625

[17] Goss G , Tsai CM , Shepherd FA , Bazhenova L , Lee JS , Chang GC , et al. Osimertinib for pretreated EGFR Thr790Met‐positive advanced non‐small‐cell lung cancer (AURA2): a multicentre, open‐label, single‐arm, phase 2 study. Lancet Oncol. 2016;17(12):1643–52. 27751847 10.1016/S1470-2045(16)30508-3

[18] Jänne PA , Yang JC , Kim DW , Planchard D , Ohe Y , Ramalingam SS , et al. AZD9291 in EGFR inhibitor‐resistant non‐small‐cell lung cancer. N Engl J Med. 2015;372(18):1689–99. 25923549 10.1056/NEJMoa1411817

[19] Mok TS , Wu YL , Ahn MJ , Garassino MC , Kim HR , Ramalingam SS , et al. Osimertinib or platinum‐Pemetrexed in EGFR T790M‐positive lung cancer. N Engl J Med. 2017;376(7):629–40. 27959700 10.1056/NEJMoa1612674 PMC6762027

[20] Yang JC , Wu YL , Schuler M , Sebastian M , Popat S , Yamamoto N , et al. Afatinib versus cisplatin‐based chemotherapy for EGFR mutation‐positive lung adenocarcinoma (LUX‐lung 3 and LUX‐lung 6): analysis of overall survival data from two randomised, phase 3 trials. Lancet Oncol. 2015;16(2):141–51. 25589191 10.1016/S1470-2045(14)71173-8

[21] Wu YL , Zhou C , Hu CP , Feng J , Lu S , Huang Y , et al. Afatinib versus cisplatin plus gemcitabine for first‐line treatment of Asian patients with advanced non‐small‐cell lung cancer harbouring EGFR mutations (LUX‐lung 6): an open‐label, randomised phase 3 trial. Lancet Oncol. 2014;15(2):213–22. 24439929 10.1016/S1470-2045(13)70604-1

[22] Kaneda T , Yoshioka H , Tamiya M , Tamiya A , Hata A , Okada A , et al. Differential efficacy of cisplatin plus pemetrexed between L858R and Del‐19 in advanced EGFR‐mutant non‐squamous non‐small cell lung cancer. BMC Cancer. 2018;18(1):6. 29291705 10.1186/s12885-017-3952-7 PMC5749021

[23] Kuo CH , Huang CH , Liu CY , Pavlidis S , Ko HW , Chung FT , et al. Prior EGFR‐TKI treatment in EGFR‐mutated NSCLC affects the allele frequency fraction of acquired T790M and the subsequent efficacy of Osimertinib. Target Oncol. 2019;14(4):433–40. 31346928 10.1007/s11523-019-00657-1

[24] Liao BC , Lin CC , Yang JC . Second and third‐generation epidermal growth factor receptor tyrosine kinase inhibitors in advanced nonsmall cell lung cancer. Curr Opin Oncol. 2015;27(2):94–101. 25611025 10.1097/CCO.0000000000000164

[25] Iwama E , Sakai K , Azuma K , Harada D , Nosaki K , Hotta K , et al. Exploration of resistance mechanisms for epidermal growth factor receptor‐tyrosine kinase inhibitors based on plasma analysis by digital polymerase chain reaction and next‐generation sequencing. Cancer Sci. 2018;109(12):3921–33. 30289575 10.1111/cas.13820 PMC6272092

[26] Campo M , Gerber D , Gainor JF , Heist RS , Temel JS , Shaw AT , et al. Acquired resistance to first‐line Afatinib and the challenges of prearranged progression biopsies. J Thorac Oncol. 2016;11(11):2022–6. 27553514 10.1016/j.jtho.2016.06.032

[27] Wu SG , Liu YN , Tsai MF , Chang YL , Yu CJ , Yang PC , et al. The mechanism of acquired resistance to irreversible EGFR tyrosine kinase inhibitor‐afatinib in lung adenocarcinoma patients. Oncotarget. 2016;7(11):12404–13. 26862733 10.18632/oncotarget.7189 PMC4914294

[28] Sequist LV , Waltman BA , Dias‐Santagata D , Digumarthy S , Turke AB , Fidias P , et al. Genotypic and histological evolution of lung cancers acquiring resistance to EGFR inhibitors. Sci Transl Med. 2011;3(75):75ra26. 10.1126/scitranslmed.3002003 PMC3132801 21430269

[29] Oxnard GR , Thress KS , Alden RS , Lawrance R , Paweletz CP , Cantarini M , et al. Association between plasma genotyping and outcomes of treatment with Osimertinib (AZD9291) in advanced non‐small‐cell lung cancer. J Clin Oncol. 2016;34(28):3375–82. 27354477 10.1200/JCO.2016.66.7162 PMC5035123

[30] Remon J , Caramella C , Jovelet C , Lacroix L , Lawson A , Smalley S , et al. Osimertinib benefit in EGFR‐mutant NSCLC patients with T790M‐mutation detected by circulating tumour DNA. Ann Oncol. 2017;28(4):784–90. 28104619 10.1093/annonc/mdx017

[31] Yang JC , Ahn MJ , Kim DW , Ramalingam SS , Sequist LV , Wc S , et al. Osimertinib in pretreated T790M‐positive advanced non‐small‐cell lung cancer: AURA study phase II extension component. J Clin Oncol. 2017;35(12):1288–96. 28221867 10.1200/JCO.2016.70.3223

[32] Viswanathan A , Pillot G , Govindan R . Lack of response to erlotinib after progression on gefitinib in patients with advanced non‐small cell lung cancer. Lung Cancer. 2005;50(3):417–8. 16129510 10.1016/j.lungcan.2005.07.004

[33] Cho BC , Im CK , Park MS , Kim SK , Chang J , Park JP , et al. Phase II study of erlotinib in advanced non‐small‐cell lung cancer after failure of gefitinib. J Clin Oncol. 2007;25(18):2528–33. 17577030 10.1200/JCO.2006.10.4166

[34] Lee DH , Kim SW , Suh C , Yoon DH , Yi EJ , Lee JS . Phase II study of erlotinib as a salvage treatment for non‐small‐cell lung cancer patients after failure of gefitinib treatment. Ann Oncol. 2008;19(12):2039–42. 18644828 10.1093/annonc/mdn423 PMC2733114

[35] Wong AS , Soong R , Seah SB , Lim SW , Chuah KL , Nga ME , et al. Evidence for disease control with erlotinib after gefitinib failure in typical gefitinib‐sensitive Asian patients with non‐small cell lung cancer. J Thorac Oncol. 2008;3(4):400–4. 18379359 10.1097/JTO.0b013e318168c801

[36] Oh IJ , Ban HJ , Kim KS , Kim YC . Retreatment of gefitinib in patients with non‐small‐cell lung cancer who previously controlled to gefitinib: a single‐arm, open‐label, phase II study. Lung Cancer. 2012;77(1):121–7. 22333554 10.1016/j.lungcan.2012.01.012

[37] Arcila ME , Oxnard GR , Nafa K , Riely GJ , Solomon SB , Zakowski MF , et al. Rebiopsy of lung cancer patients with acquired resistance to EGFR inhibitors and enhanced detection of the T790M mutation using a locked nucleic acid‐based assay. Clin Cancer Res. 2011;17(5):1169–80. 21248300 10.1158/1078-0432.CCR-10-2277 PMC3070951

[38] Sabaawy HE . Genetic heterogeneity and clonal evolution of tumor cells and their impact on precision cancer medicine. J Leukem. 2013;1(4):1000124. 10.4172/2329-6917.1000124 PMC3927925 24558642

[39] Tseng JS , Su KY , Yang TY , Chen KC , Hsu KH , Chen HY , et al. The emergence of T790M mutation in EGFR‐mutant lung adenocarcinoma patients having a history of acquired resistance to EGFR‐TKI: focus on rebiopsy timing and long‐term existence of T790M. Oncotarget. 2016;7(30):48059–69. 27384480 10.18632/oncotarget.10351 PMC5217000

[40] Dagogo‐Jack I , Shaw AT . Tumour heterogeneity and resistance to cancer therapies. Nat Rev Clin Oncol. 2018;15(2):81–94. 29115304 10.1038/nrclinonc.2017.166

[41] Zheng C , Li X , Ren Y , Yin Z , Zhou B . Coexisting EGFR and TP53 mutations in lung adenocarcinoma patients are associated with COMP and ITGB8 upregulation and poor prognosis. Front Mol Biosci. 2020;7:30. 32175330 10.3389/fmolb.2020.00030 PMC7056714

[42] Nayak L , Lee EQ , Wen PY . Epidemiology of brain metastases. Curr Oncol Rep. 2012;14(1):48–54. 22012633 10.1007/s11912-011-0203-y

[43] Rangachari D , Yamaguchi N , VanderLaan PA , Folch E , Mahadevan A , Floyd SR , et al. Brain metastases in patients with EGFR‐mutated or ALK‐rearranged non‐small‐cell lung cancers. Lung Cancer. 2015;88(1):108–11. 25682925 10.1016/j.lungcan.2015.01.020 PMC4355240

[44] Soria JC , Ohe Y , Vansteenkiste J , Reungwetwattana T , Chewaskulyong B , Lee KH , et al. Osimertinib in untreated EGFR‐mutated advanced non‐small‐cell lung cancer. N Engl J Med. 2018;378(2):113–25. 29151359 10.1056/NEJMoa1713137

